# SCF^FBXW7α^ modulates the intra-S-phase DNA-damage checkpoint by regulating Polo like kinase-1 stability

**DOI:** 10.18632/oncotarget.2021

**Published:** 2014-05-27

**Authors:** Servando Giráldez, Joaquín Herrero-Ruiz, Mar Mora-Santos, Miguel Á. Japón, Maria Tortolero, Francisco Romero

**Affiliations:** ^1^ Departamento de Microbiología, Facultad de Biología, Universidad de Sevilla. Apartado de correos 1095. 41080-Sevilla, Spain; ^2^ Instituto de Biomedicina de Sevilla (IBIS), Hospital Universitario Virgen del Rocío/CSIC/Universidad de Sevilla and Departamento de Anatomía Patológica, Hospital Universitario Virgen del Rocío, 41013 Sevilla, Spain

**Keywords:** PLK1, FBXW7, intra-S-phase, DNA-damage, proteasome, protein degradation

## Abstract

The intra-S-checkpoint is essential to control cell progression through S phase under normal conditions and in response to replication stress. When DNA lesions are detected, replication fork progression is blocked allowing time for repair to avoid genomic instability and the risk of cancer. DNA replication initiates at many origins of replication in eukaryotic cells, where a series of proteins form pre-replicative complexes (pre-RCs) that are activated to become pre-initiation complexes and ensure a single round of replication in each cell cycle. PLK1 plays an important role in the regulation of DNA replication, contributing to the regulation of pre-RCs formation by phosphorylating several proteins, under both normal and stress conditions. Here we report that PLK1 is ubiquitinated and degraded by SCF^FBXW7α^/proteasome. Moreover, we identified a new Cdc4 phosphodegron in PLK1, conserved from yeast to humans, whose mutation prevents PLK1 destruction. We established that endogenous SCF^FBXW7α^ degrades PLK1 in the G1 and S phases of an unperturbed cell cycle and in S phase following UV irradiation. Furthermore, we showed that FBXW7α overexpression or UV irradiation prevented the loading of proteins onto chromatin to form pre-RCs and, accordingly, reduced cell proliferation. We conclude that PLK1 degradation mediated by SCF^FBXW7α^ modulates the intra-S-phase checkpoint.

## INTRODUCTION

*FBXW7* is a tumor suppressor gene that is frequently inactivated in different types of cancer, including breast cancer, colon cancer and leukemia [1]. FBXW7 protein is a member of the F-box family of proteins, components of Skp1, Cul1, and F-box protein (SCF) ubiquitin ligase complexes. F-box proteins are responsible for recruiting specific substrates for ubiquitination and degradation [2]. FBXW7 targets several oncoproteins for proteolysis, such as cyclin E, c-Jun, c-Myc, Mcl-1 or Notch [3]. Mammalian cells contain three FBXW7 isoforms, FBXW7α, FBXW7β and FBXW7γ, that are produced by alternative splicing and localize to the nucleoplasm, cytoplasm and nucleolus, respectively [4, 5]. FBXW7α is the most highly expressed and stable FBXW7 isoform and expression levels of this protein do not vary significantly during the cell cycle [4, 6]. The *FBXW7α* transcript is ubiquitously expressed in all human tissues and is also induced by the p53 tumor suppressor in response to DNA damage [7, 8].

The FBXW7α protein contains several protein-protein interaction domains, including a dimerization domain, an F-box domain that recruits the SCF core complex, and eight WD40 repeats that form a β-propeller binding pocket [9-11]. Notably, it has been shown that WD40 β-propellers function as ubiquitin-binding domains and that ubiquitin interaction by FBXW7 promotes its auto-ubiquitination and turnover [12]. However, the importance of FBXW7α dimerization is still not entirely clear, but it has been proposed to increase the ubiquitination efficiency of low affinity substrates [11]. More recently, it has been reported that Pin1, a prolyl isomerase, interacts with FBXW7α in a phosphorylation-dependent manner and promotes FBXW7α auto-ubiquitination and protein degradation by disrupting FBXW7α dimerization, suggesting that inhibition of Pin1 could upregulate the expression of FBXW7α to retard the growth of human tumor cells [13].

FBXW7 binds to substrates via its WD40 domain located in the carboxy-terminus of the protein, which interacts with a phosphothreonine-containing motif, known as CPD (Cdc4 phosphodegron), in the substrates [14, 15]. SCF^FBXW7^ activity is regulated by various factors, among which are an active neddylation system [16], Pin1 and/or PP2A [17], and the deubiquitinating enzyme USP28 [18]. Interestingly, USP28 dissociates from FBXW7α in response to UV irradiation, providing a mechanism for how FBXW7α-mediated degradation of c-Myc is enhanced upon DNA damage [19]. Finally, FBXW7α-dependent substrate ubiquitination is also dependent on upstream signaling pathways, including the PI3K/Akt/GSK3β pathway [20], the ATM/ATR pathway upon induction of DNA damage [21], and the Ras signaling pathway [22].

Polo-like kinase 1 (PLK1) is a highly conserved serine/threonine kinase that plays a key role in eukaryotic cell division [23]. Expression of PLK1 increases in S phase and peaks during mitosis. PLK1 mediates many mitotic events, including entry into mitosis, centrosome maturation, assembly of the bipolar spindle, sister chromatid splitting, activation of the Anaphase-Promoting Complex/Cyclosome (APC/C), and exit from mitosis with the initiation of cytokinesis [24]. In addition, PLK1 has a plethora of roles being implicated in microtubule dynamics, DNA replication, chromosome dynamics, p53 regulation and recovery from the G2 DNA damage checkpoint [25]. Furthermore, PLK1 is degraded by the APC/C^CDH1^ from late anaphase, for the proper control of mitotic exit and cytokinesis, to the entry of cells into the G1 phase [26], and also after DNA-damage in G2 [27].

The transfer of genetic information with high fidelity from parent to daughter cells is one of the most important tasks of the cell cycle. Besides mitosis, where the replicated chromosomes are segregated, DNA replication during S phase is an essential stage for the maintenance of genome integrity. For the initiation of DNA replication, a series of proteins are assembled on each replication origin. Origin recognition complex (ORC) 1-6 subunits, which bind to the replication origins, and mini-chromosome maintenance (MCM) complex 2-7 subunits, which are loaded onto the origins, depending on Cdt1 and Cdc6, are involved in the formation of the pre-replicative complexes (pre-RCs). The MCM complex is the DNA helicase that plays a central role in the progression of replication forks. Later, other proteins are loaded onto pre-RCs to form pre-initiation complexes (pre-ICs), and two classes of kinases, DDK (Dbf4-dependent kinase) and CDK (cyclin-dependent kinase), play important roles in the assembly of pre-ICs and ensure that only a single round of DNA replication takes place in each cell cycle. Under unperturbed conditions, replication starts from a subset of these origins. Two replication forks move in opposite directions from each origin behind the CMG complex, formed by Cdc45, MCM and GINS, which unwinds the DNA, providing single-stranded DNA templates for the polymerases to duplicate. When progression of DNA replication forks is hindered, activation of checkpoint pathways temporarily halts the cell cycle progression, giving the cells time to solve replication problems before entering into mitosis (for review, see [28]).

Several reports associate PLK1 with DNA replication. In this regard, PLK1 depletion in the long term slows proliferation apparently due to attenuated progression through the S phase [29]. In addition, PLK1 interacts with MCM2, MCM3, MCM7 and ORC2, and phosphorylates Hbo1, regulating pre-RC loading of MCM2 and MCM6 [30-32]. Moreover, PLK has also been implicated in replication under stressful conditions. It has been shown that the *Xenopus* PLK homolog, Plx1, phosphorylates Claspin, one of the proteins that mediates the interaction between the MCM helicase and DNA polymerases, causing its dissociation from chromatin and resulting in the inactivation of the replication checkpoint kinase Chk1 after a prolonged checkpoint arrest [33]. The PLK homolog in budding yeast, Cdc5, is also required for the adaptation, that is the resumption of the cell cycle in the presence of a single unrepaired DSB after a prolonged arrest [34]. Furthermore, a novel role has been suggested for PLK1 in maintenance of genomic integrity by promoting DNA replication under conditions of stress [35]. Together, these findings indicate that PLK has an inhibitory effect on the checkpoint response.

In this study, we identify PLK1 as a novel SCF^FBXW7α^ substrate and show how it is degraded in G1- and S-phase-arrested cells, and in response to DNA damage. Additionally, we demonstrate that PLK1 degradation impedes the formation of pre-RCs and, following DNA damage, avoids cell proliferation.

## RESULTS

### Identification of FBXW7α-interacting proteins

**Figure 1:**
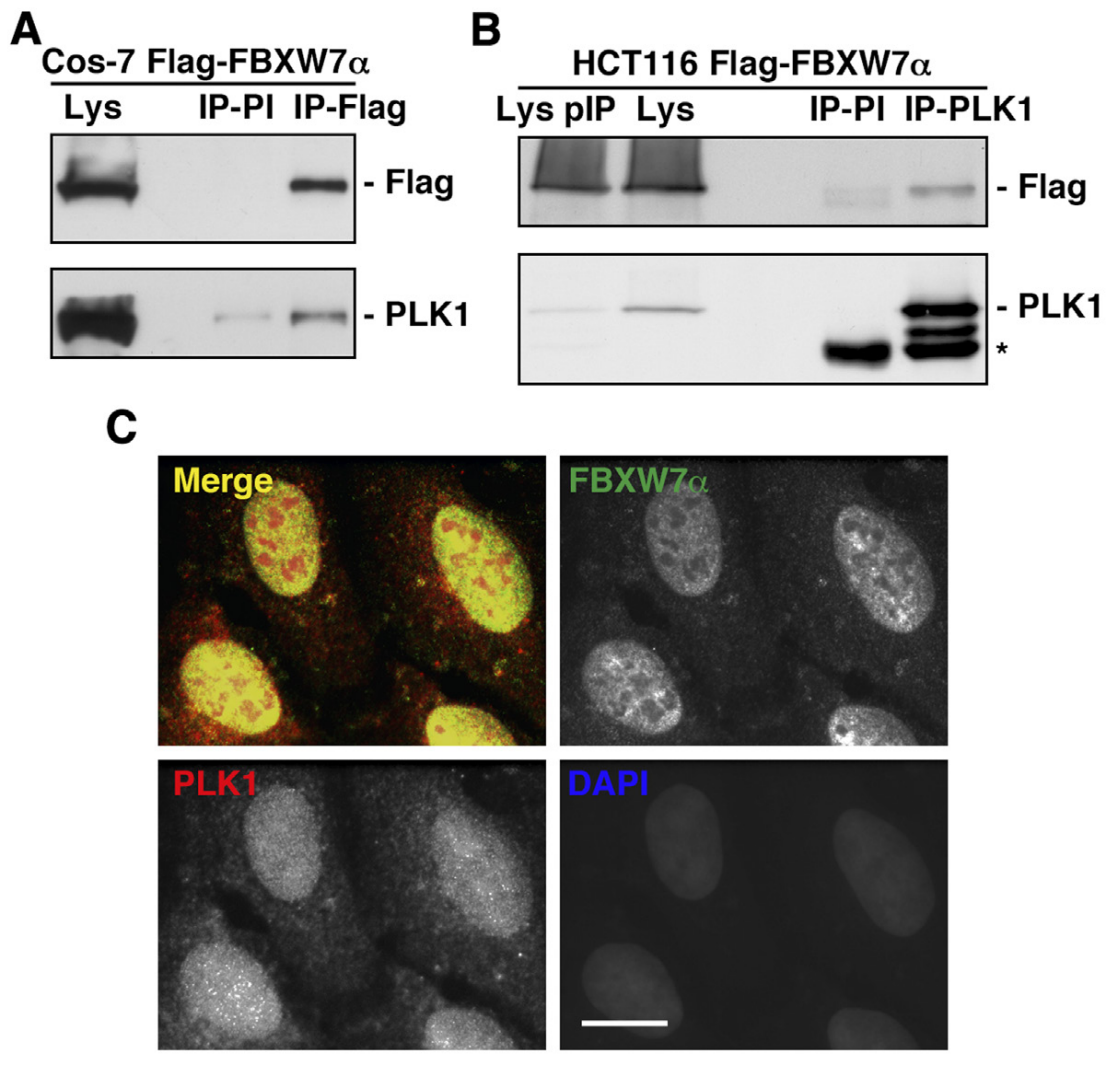
FBXW7α and PLK1 interact in the nuclei of mammalian cells (A) Cos-7 cells were transiently transfected with pCDNA3.1-Flag-FBXW7α and nuclear extracts immunoprecipitated with anti-Flag monoclonal antibody or normal mouse serum (PI). Immunoprecipitates materials were analyzed by Western blotting. Lys: nuclear extracts from Cos-7 transfected cells. (B) Whole cell extracts from HCT116 transfected cells were used to immunoprecipitate PLK1, and complexes were analyzed by immunoblotting. IP-PI: immunoprecipitation with normal mouse serum. Lys and Lys pIP: whole cell extracts from HCT116 transfected cells before (Lys) and after (Lys pIP) PLK1 immunoprecipitation. Asterisk indicates IgG heavy chains. (C) U2OS cells were stained for FBXW7α, PLK1 and DNA. In the merge, FBXW7α staining is shown in green, PLK1 in red, and DAPI in blue. Bar, 10μm.

To identify new SCF^FBXW7α^ substrates, we first searched for FBXW7α binding proteins by FBXW7α immunoprecipitation and tandem mass spectrometry assays (MS/MS). We transfected several cell lines with Flag-FBXW7α and confirmed by Western blot and immunofluorescence experiments its nuclear localization, as described for endogenous FBXW7α (supplementary Figs S1A and S1B) [6]. Duplicate MS/MS analysis of Flag-FBXW7α immunoprecipitation from Cos-7 nuclear extracts resulted in the identification of PLK1 as a novel FBXW7α-interacting protein. To further validate the authenticity of these results, we confirmed the presence of PLK1 within the FBXW7α immunocomplex using Western blot analysis (Fig 1A). In addition, we performed reciprocal immunoprecipitations using HCT116 transfected cells and monoclonal antibodies to PLK1. Immunoprecipitated proteins were resolved using SDS-PAGE and the band correlating to PLK1 was identified by Western blot (Fig 1B). Consistent with the results from the interaction assays, we localized by immunofluorescence both endogenous FBXW7α and PLK1 in the nuclei of U2OS cells (Fig 1C). Taken together, our findings show that FBXW7α and PLK1 associate in intact cells.

### SCF^FBXW7α^ targets PLK1 for ubiquitination

**Figure 2:**
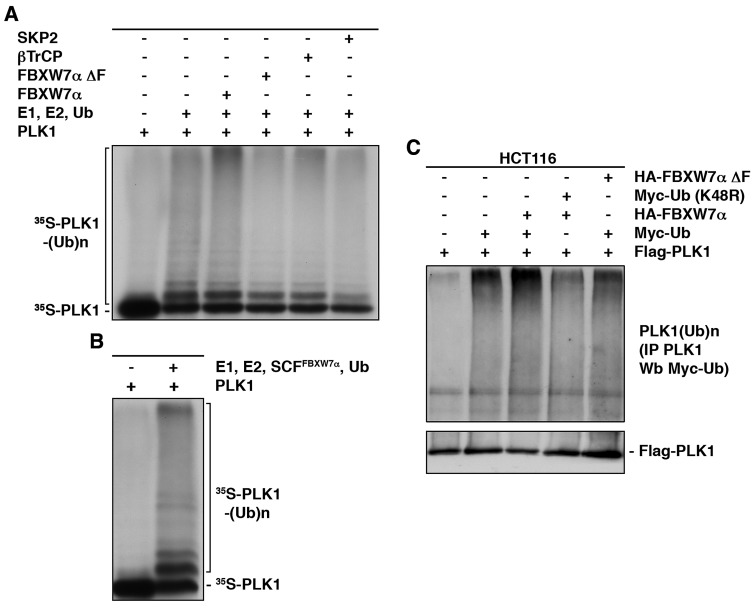
SCF^FBXW7α^ ubiquitinates PLK1 (A) *In vitro* ubiquitin ligation assay of ^35^S labeled *in vitro*-transcribed/translated PLK1 was conducted in the presence or absence of the following products: cold *in vitro*-transcribed/translated SKP2, βTrCP, FBXW7αΔF or FBXW7α, E1 (His_6_-ubiquitin activating enzyme), E2 (His_6_-UbcH3 and UbcH5a) and Ub (ubiquitin). Samples were incubated at 30°C for 1h. The bracket on the left side marks a ladder of bands corresponding to poly-ubiquitinated PLK1. (B) The experiment was performed as in (A) except that the unlabeled F-box protein was substituted by a recombinant SCF^FBXW7α^ complex expressed in Sf21 insect cells. (C) HCT116 cells were transfected with plasmids encoding the indicated proteins, and treated with LLnL for 4h before harvesting. Extracts were prepared as indicated in the Materials and Methods and poly-ubiquitinated PLK1 visualized after Western blots of the PLK1 immunoprecipitations.

To investigate whether SCF^FBXW7α^ promotes PLK1 ubiquitination, we performed both *in vitro* and *in vivo* ubiquitination assays. Figure 2A shows an increment of the *in vitro*-transcribed/translated PLK1 ubiquitination in the presence of FBXW7α. Other F-box proteins, βTrCP or SKP2, or FBXW7αΔF, a dominant-negative variant of FBXW7α lacking the F-box domain [36] which couples F-box proteins to the SCF complexes, were unable to trigger a significant ubiquitination of PLK1. Similar results were obtained when we used a recombinant SCF^FBXW7α^ complex expressed in Sf21 insect cells (Fig 2B). In agreement with our findings *in vitro*, the *in vivo* results showed that FBXW7α promotes the ubiquitination of PLK1 in HCT116 transfected cells. As shown in Figure 2C, Flag-PLK1 immunoprecipitations presented an increment in the levels of poly-ubiquitinated forms when cells were co-transfected with FBXW7α and Myc-Ubiquitin, but not with FBXW7αΔF or an ubiquitin mutant that blocks the formation of the poly-ubiquitin chains (Myc-Ub (K48R)). These results show that SCF^FBXW7α^ targets PLK1 for ubiquitination.

### SCF^FBXW7α^ promotes proteasomal turnover of PLK1 in the G1 and S phases of the cell cycle

**Figure 3:**
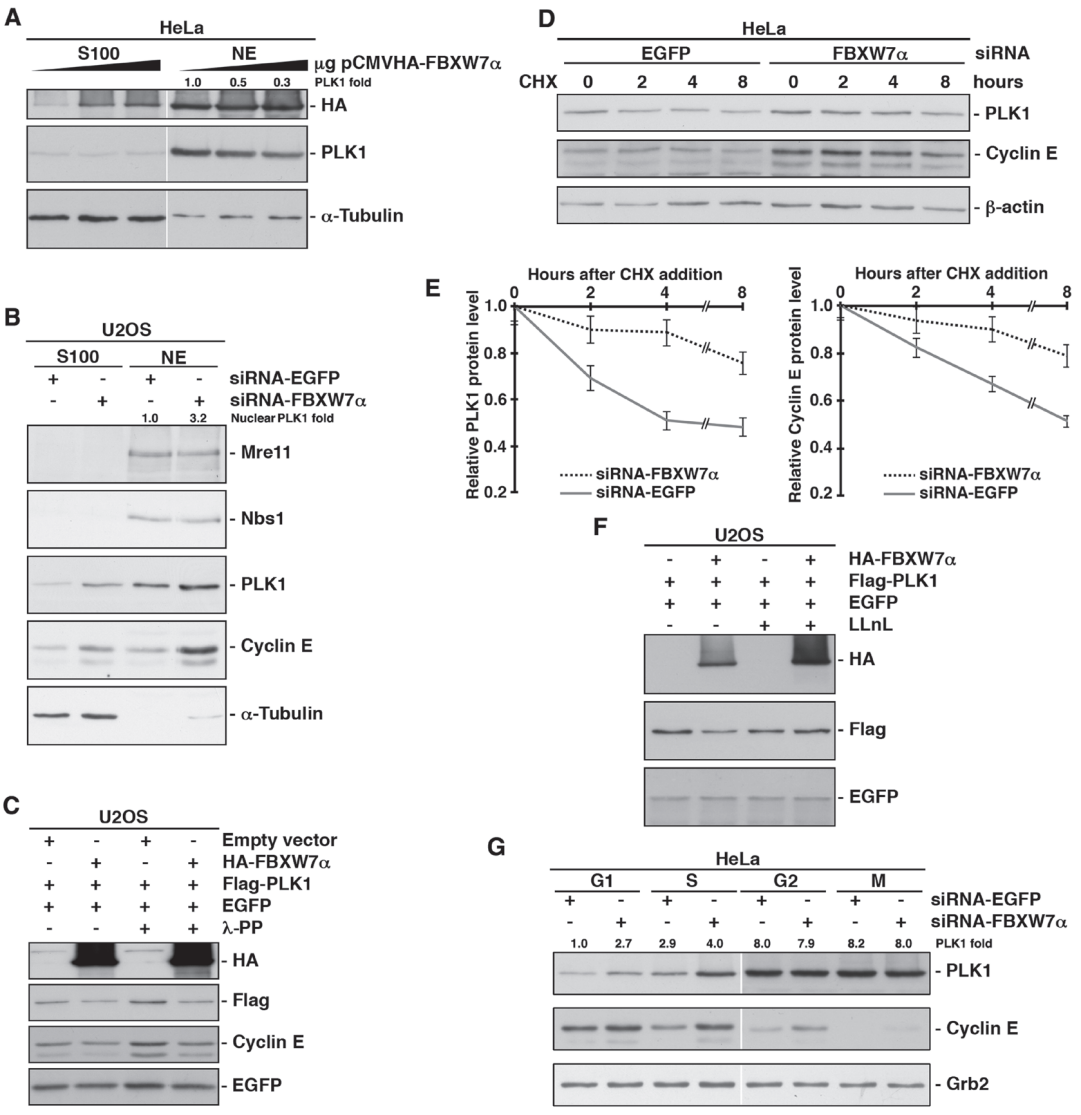
SCF^FBXW7α^ mediates PLK1 proteasomal degradation in the G1 and S phases (A) HeLa cells were transfected with increasing amount of pCMVHA-FBXW7α and, 18h later, cytosolic (S100) and nuclear extracts (NE) were subjected to Western blot. (B) U2OS cells interfered with siRNA-FBXW7α or siRNA-EGFP as a control, were used to prepare cytosolic (S100) and nuclear extracts (NE), and fractions were analyzed for the presence of different proteins as indicated. (C) Whole cell extracts from U2OS cells transfected with plasmids encoding the indicated proteins were treated or not with lambda phosphatase (λ-PP), migrated, electroblotted and probed with different antibodies. (D) HeLa cells were interfered with EGFP- or FBXW7α-siRNA and, after 48h, cycloheximide (CHX) was added to the medium and cells were collected at the indicated times. Extracts were analyzed by Western blot. (E) Quantification of PLK1 and cyclin E protein levels presented in (D) using the ImageJ software. Error bars represent the S.D. (n=3). (F) U2OS cells were transiently transfected with plasmids encoding the indicated proteins and, treated or not with LLnL for 4h before harvesting. Lysates were analyzed by Western blotting. (G) HeLa cells were interfered with the indicated siRNA and arrested in the different phases of the cell cycle. Extracts were blotted with different antibodies. Grb2 expression was used as a loading control. The quantitative fold change in PLK1 was determined relative to the loading control.

To examine whether SCF^FBXW7α^ was responsible for PLK1 degradation, we first overexpressed FBXW7α in HeLa cells and found that increasing amounts of FBXW7α correlated with decreasing amounts of endogenous PLK1 (Fig 3A). We then down-regulated FBXW7α expression using an established siRNA [37] to test whether FBXW7α depletion stabilizes PLK1. Knock-down of FBXW7α was confirmed by expressing exogenous protein (supplementary Fig S2A), due to the inefficient detection of endogenous FBXW7α using commercial antibodies. Cytosolic (S100 fraction) and nuclear extracts interfered with siRNA-FBXW7α were analyzed for the presence of PLK1 in U2OS cells, and an important increase of PLK1 was observed in nuclear extracts (Fig 3B). Consistent with previous reports, cyclin E, a known target of FBXW7α [38, 39], was also stabilized by depletion of FBXW7α, but not Mre11, Nbs1 or α-Tubulin that acted as controls. To avoid possible indirect effects on transcriptional regulation of PLK1, we analyzed the effect of the overexpression of FBXW7α on ectopically expressed PLK1 using a constitutive heterologous promoter. As compared with empty vector, ectopic FBXW7α also reduced the levels of PLK1, including after lambda phosphatase treatment, to discount the possibility that PLK1 was phosphorylated (Fig 3C).

To demonstrate that the loss of PLK1 in the presence of FBXW7α was due to proteolysis, we carried out PLK1 half-life determinations after inhibition of protein synthesis by treatment with cycloheximide. HeLa cells interfered with siRNA-FBXW7α showed a longer PLK1 half-life than when cells were interfered with siRNA-EGFP (Figs 3D and 3E). As an internal control we analyzed the cyclin E half-life, obtaining similar results. In the same way, when we performed the reverse experiment using exogenous PLK1 and FBXW7α, we reduced the PLK1 half-life (supplementary Figs S2B and S2C). The FBXW7α-mediated degradation of PLK1 was alleviated by the inclusion of a proteasome inhibitor in the experiment, strongly suggesting that the ubiquitinated PLK1 is degraded by the 26S proteasome (Fig 3F).

As protein degradation constitutes the major post-translational control of cell cycle regulators, we examined whether PLK1 degradation occurs in a cell cycle phase-specific manner. HeLa cells were interfered with siRNA-FBXW7α and arrested in the different phases of the cell cycle (supplementary Fig S2D), and we found that the amount of PLK1 augmented both in G1 and S, but not in G2 or M phases (Fig 3G). Complementary results were found when we overexpressed FBXW7α, namely, FBXW7α caused the PLK1 diminution in both butyrate (G1 phase) and hydroxyurea (S phase) treated cells (supplementary Fig S2E). Taken together, our results clearly show that SCF^FBXW7α^ promotes proteasomal degradation of PLK1 in the G1 and S phases of a normal cell cycle.

### SCF^FBXW7α^ regulates PLK1 levels in response to UV irradiation

**Figure 4:**
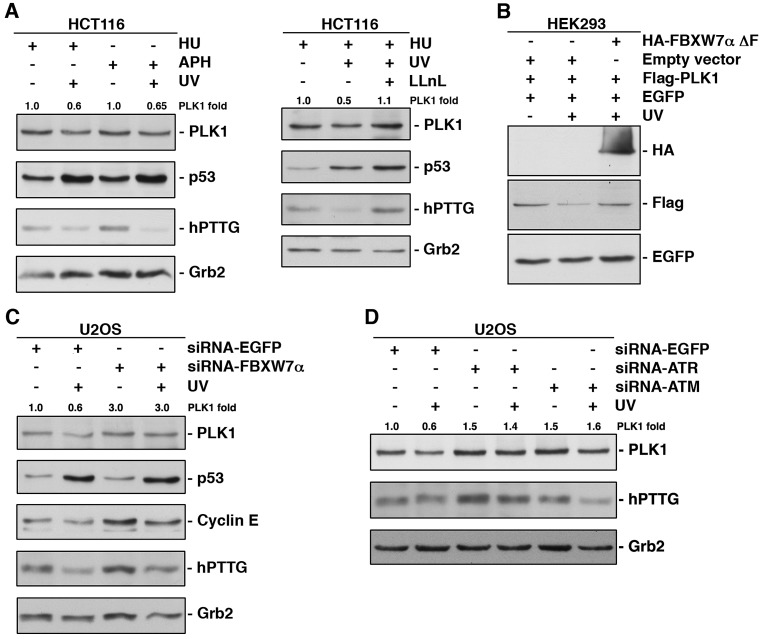
UV irradiation accelerates PLK1 degradation in S phase of the cell cycle via SCF/^FBXW7α^proteasome (A) HCT116 cells arrested in S phase using hydroxyurea (HU) or aphidicolin (APH) were irradiated (30J/m^2^) or not, and extracts analyzed by Western blot. In the right panel, cells were treated with LLnL 30 min before irradiation. p53 and hPTTG were used as controls whose degradation is avoided or induced by UV. Grb2 expression was used as a loading control. (B) HEK293 cells were transfected and subjected or not to UV irradiation before harvesting. Extracts were analyzed by Western blot. (C) U2OS cells were interfered with EGFP- or FBXW7α-siRNA, arrested in S phase with HU and irradiated where indicated. Extracts were analyzed by Western blot. Cyclin E and hPTTG were used as a positive and a negative control, respectively, of FBXW7 depletion. (D) U2OS cells interfered with the indicated siRNA and arrested in S phase (HU) were irradiated or not 4h before harvesting, and proteins immunoblotted with different antibodies. The quantitative fold change in PLK1 was determined relative to the loading control.

It has been published that PLK1 is degraded by the APC/C^CDH1^/proteasome in response to DNA damage in G2, avoiding entry into mitosis and instead initiating DNA repair [27]. On the other hand, it is also known that PLK1 has an important role during S phase [40, 41]. In this study, we have demonstrated that SCF^FBXW7α^ mediates PLK1 proteolytic degradation in S. However, little is known about the potential effects of DNA damage on the role of PLK1 in S phase. Therefore, we decided to investigate whether PLK1 might be degraded after DNA damage in S phase and whether SCF^FBXW7α^ would be involved in this degradation. To this end, we analyzed the PLK1 protein level in several cell lines synchronized in S phase (by double thymidine block followed by a 4h release, or by treatment with hydroxyurea or aphidicolin), before and after UV irradiation. In Figure 4A (and supplementary Figs S3A-C) a significant decrease of PLK1 was observed following UV treatment, and this decrease could be reversed using a proteasome inhibitor. In addition, FBXW7αΔF was able to avoid PLK1 degradation after UV treatment (Fig 4B), indicating that SCF^FBXW7α^ is also responsible for the regulation of PLK1 levels in response to DNA damage. In fact, silencing of FBXW7α reduced the PLK1 turnover after UV irradiation in S phase compared with mock treated cells (Fig 4C).

The ATR and ATM protein kinases are recruited to defective replication forks or to sites of DNA damage, and their homologs are thought to initiate the DNA damage response in all eukaryotes [42]. To determine whether these kinases are involved in PLK1 degradation in S phase after UV irradiation, we carried out down-regulation experiments in this phase and detected PLK1 levels by Western blot before and after UV treatment. As shown in Figure 4D, both ATM- and ATR-depleted cells prevented the degradation of PLK1 after UV irradiation in a similar way to the knock-down of FBXW7α (Fig 4C). Comparable results were obtained after treatment with caffeine, an inhibitor of ATM and ATR kinases, or UCN-01, which inhibits the ATR-Chk1 pathway after UV treatment (supplementary Fig S3D). Overall, these results show that SCF^FBXW7α^ mediates PLK1 degradation induced by UV irradiation and suggest the involvement of ATM/ATR in this response.

### Identification of a Cdc4 phosphodegron in PLK1

**Figure 5:**
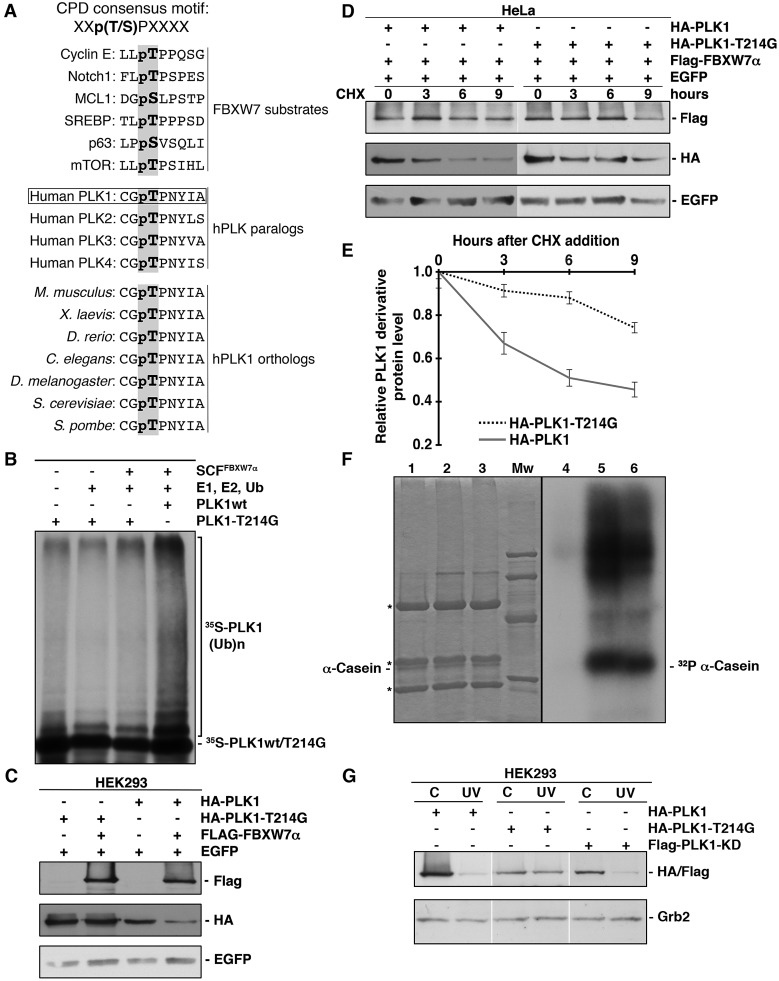
PLK1 possesses a functional CPD motif (A) Alignment of some CPDs from different FBXW7 substrates and comparison of the CPD-like motif identified in human PLK1 (residues 212 to 219) with other human PLKs and PLK1 orthologs. X refers to any residue other than K or R. pT in bold in human PLK1 (residue 214) is the putative phosphothreonine in CPDs. (B) *In vitro* ubiquitin ligation assay of ^35^S labeled *in vitro*-transcribed/translated PLK1-T214G was conducted in the presence or absence of the following products: recombinant SCF^FBXW7α^ complex expressed in Sf21 insect cells, E1 (His_6_-ubiquitin activating enzyme), E2 (His_6_-UbcH3 and UbcH5a) and Ub (ubiquitin). ^35^S-PLK1wt was used as a positive control. Samples were incubated at 30°C for 1h. The bracket on the right side marks a ladder of bands corresponding to poly-ubiquitinated PLK1. (C) HEK293 cells were transiently transfected with plasmids encoding the indicated proteins, and extracts analyzed by Western blot. (D) HeLa cells were transfected with plasmids encoding the indicated proteins and, after 48h, CHX was added to the medium and cells were collected at the indicated times. Whole cell extracts were analyzed by Western blot. (E) Protein levels from (D) were quantified using the ImageJ software. Error bars represent the S.D. (n=3). (F) Anti-HA immunoprecipitates from lysates of HCT116 cells transfected with pCMVHA (lanes 1 and 4), pCMVHA-PLK1 (lanes 2 and 5) and pCMVHA-PLK1-T214G (lanes 3 and 6) were incubated with α-casein dephosphorylated, (γ-^32^P) ATP and a kinase buffer as described in Materials and Methods. Reactions were analyzed by SDS-PAGE, stained with Coomassie Blue (lanes 1-3), and autoradiographed (lanes 4-6). Mw: molecular mass markers (95, 66, 45 and 31 kDa). Asterisks indicate IgG heavy and light chains. (G) PLK1 (and derivatives) transfected cells were irradiated (30J/m^2^) or not and lysates subjected to Western blotting. Grb2 expression was used as a loading control.

Several reports have described that the CPD recognized by FBXW7α contains the motif L/I-L/I/P-pT-P-X-X-X-X (where X refers to any residue other than K or R) [14, 36, 43]. However, other authors have established a slightly different motif, (L)-X-pT/pS-P-(P)-X-pS/pT/E/D (where X is any amino acid) (revised in [15]). With these data in mind, we analyzed the PLK1 protein sequence and found a single non-canonical CPD motif between residues 212 and 219, C-G-T-P-N-Y-I-A. This CPD-like sequence is almost completely conserved in other human PLK family members, as well as, in PLK1 orthologs from other species (Fig 5A). To ascertain whether this sequence is a functional CPD, we mutated threonine 214 to glycine. We proved that this mutant was unable to be ubiquitinated by FBXW7α *in vitro* (Fig 5B) and degraded in transfected cells (Fig 5C). Furthermore, when we overexpressed FBXW7α the half-life of PLK1-T214G was longer than the half-life of wild-type (Figs 5D and 5E), indicating that threonine 214 is involved in the regulation of PLK1 stability. Given that threonine 214 is found within the PLK1 kinase domain, we performed an *in vitro* kinase assay using dephosphorylated α-casein as a substrate. This assay confirmed that the PLK1-T214G mutant still retained its kinase activity (Fig 5F), suggesting that the overall structure of this mutant protein remains largely intact. Finally, we analyzed the effect of UV irradiation on the degradation of the PLK1-T214G mutant. We found that point mutation of threonine 214 clearly prevented the PLK1 degradation induced by UV, while other point mutant (PLK1-KD) was degraded (Fig 5G). Therefore, our findings show that PLK1 contains a CPD motif that promotes PLK1 degradation following UV irradiation and that this motif is highly conserved from yeast to humans.

### PLK1 degradation avoids the cell proliferation after DNA damage in S-phase

**Figure 6:**
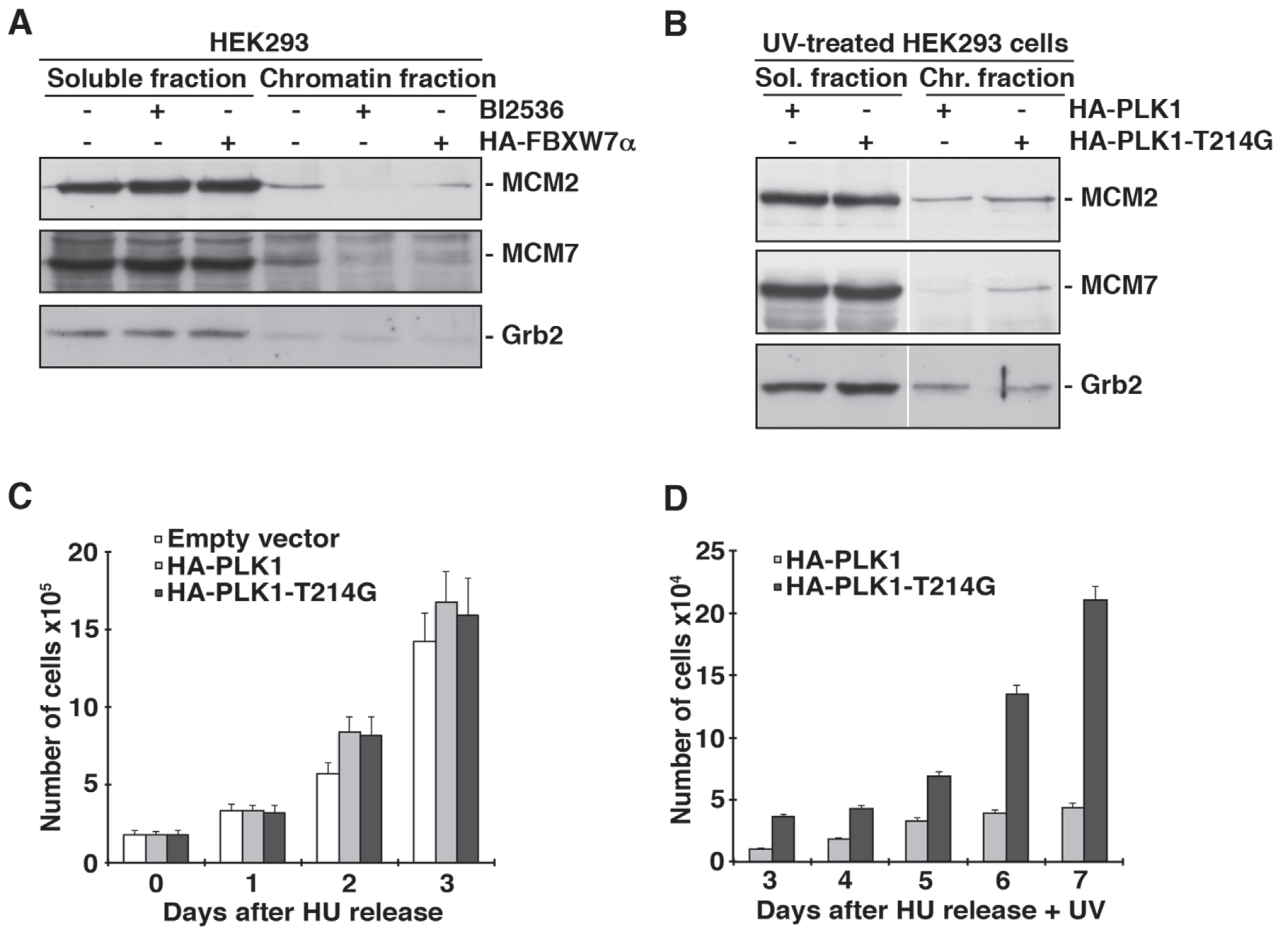
PLK1 degradation by SCF^FBXW7α^ reduces pre-RC formation and cell proliferation (A) HEK293 cells were transfected with pCMVHA-FBXW7α for 18h or treated with BI2536 for 8h, and subjected to chromatin fractionation as described in Materials and Methods. Fractions were treated with λ-PP and blotted with the indicated antibodies. (B) HEK293 cells were transfected with pCMVHA-PLK1 and pCMVHA-PLK1-T214G, irradiated and subjected to chromatin fractionation as described. Extracts were treated with λ-PP and analyzed by Western blot. (C) HeLa cells were transfected with pCMVHA (empty vector), pCMVHA-PLK1 and pCMVHA-PLK1-T214G for 18h, arrested in S phase with HU for 24h and, after releasing, trypan blue-excluding cells were counted using a hemocytometer at different time points. The analysis began with equal numbers of the differently transfected cells. Error bars** represent the S.D. (n=3). (D) Transfected cells were treated as in (C) but just after HU release, cells were UV irradiated. Viable cells were counted, starting three days after release, for 5 days.

In this study, we have shown that PLK1 is ubiquitinated by SCF^FBXW7α^ and degraded in the G1 and S phases of the cell cycle and in response to DNA damage. To elucidate the role of this ubiquitination and degradation, we analyzed the involvement of PLK1 in the intra-S-phase checkpoint. Firstly, we tested the possibility that FBXW7α overexpression could reduce the amount of MCM proteins loaded onto chromatin, as mediated by PLK1. Figure 6A shows that the chromatin loading of MCM2 and MCM7 in FBXW7α-transfected HEK293 cells is less than that found in non-transfected control cells. Similar results were obtained from cells treated with BI2536, a potent and selective PLK1 inhibitor. Moreover, we observed an important diminution of MCM proteins in the chromatin fraction of UV irradiated HEK293 cells (data not shown). Next, we examined the distribution of MCM proteins after UV irradiation of HEK293 cells transfected with PLK1 or PLK1-T214G. Cells transfected with the non-degradable PLK1 mutant conserved more MCM proteins on chromatin than cells transfected with wild-type PLK1, indicating that the PLK1 degradation mediated by SCF^FBXW7α^ impedes the formation of pre-RCs (Fig 6B). To determine whether PLK1-T214G altered cell proliferation following UV treatment, we transfected HeLa cells with PLK1, PLK1-T214G or empty vector, and then arrested cells in S-phase by the addition of hydroxyurea. After release, cells were irradiated or not and cell proliferation was quantified. As shown in Figure 6C (and supplementary Fig S4A), the three transfected cell lines had a similar proliferation pattern. However, after UV irradiation, the presence of PLK1-T214G mutant in HeLa cells accelerated cell proliferation (Fig 6D and supplementary Fig S4B). Similar results were obtained in U2OS transfected cells (data not shown). Therefore, we can conclude that PLK1 degradation by SCF^FBXW7α^ avoids cell proliferation after DNA damage in the S-phase of the cell cycle.

## DISCUSSION

Cancer is the consequence of intra- and extracellular signaling network dysregulation that derives from the activation of oncogenes or inactivation of tumor suppressor genes. Cancer cells exhibit altered signaling pathways with adaptations that overcome cellular safeguards that prevent oncogenic transformation. Both PLK1 and FBXW7α are factors involved in tumorigenesis. PLK1 is considered a proto-oncogene, whose overexpression is often observed in tumor cells and FBXW7α is a tumor suppressor whose mutation occurs in multiple neoplasms. Overexpression of PLK1 has been identified in samples taken from patients with lung, breast, colon, pancreas, prostate and ovary tumors, and approximately 6% of all primary human tumors harbor mutations in *FBXW7α*, with the greatest mutation rates found in cholangio-carcinoma and T-cell acute lymphoblastic leukemia [1, 44]. The misregulated degradation of tumor suppressors or oncoproteins can also drive tumorigenesis. Accordingly, an overexpressed (or underexpressed) F-box protein can function as an oncoprotein or as a tumor suppressor depending on whether their substrates are tumor suppressors or oncoproteins, respectively. Here we show that PLK1 interacts with FBXW7α *in vivo*, is specifically ubiquitinated both *in vitro* and *in vivo* by SCF^FBXW7α^ and is degraded via the proteasome. This degradation occurs in control conditions and after UV irradiation. These results led us to propose that, as for other SCF^FBXW7α^ substrates, such c-Myc, c-Jun, cyclin E and Notch [3], FBXW7α is also acting as a tumor suppressor, avoiding excessive cell proliferation in unstressed conditions and after DNA damage via control of PLK1. Down-regulation of endogenous PLK1 in several human cell lines significantly decreases cell proliferation and migrating ability, and overexpression of PLK1 in NIH3T3 cells induces oncogenic transformation [45, 46]. Our proliferation experiments in S phase after UV irradiation using PLK1-transfected cells versus transfected cells with a non-degradable SCF^FBXW7α^ PLK1 point mutant (PLK1-T214G) showed that PLK1 degradation is necessary for prolonging cell proliferation arrest after DNA damage.

During undisturbed DNA replication, PLK1 promotes pre-RC loading via phosphorylation of Hbo1. When DNA replication is under stress, checkpoint activation causes active replication forks to stall and PLK1 phosphorylates ORC2, promoting the maintenance of pre-RC on dormant replication origins [30-32]. Importantly, our findings show that the monitoring performed by PLK1 on DNA replication is regulated by PLK1 degradation via SCF^FBXW7α^. These results are supported by the fact that the PLK1-T214G mutant was still able to promote MCM loading onto chromatin after UV irradiation, and that, as mentioned above, PLK1-T214G transfected cells reduced the cell cycle arrest after DNA damage. Interestingly, the new CPD motif identified in PLK1 is phylogenetically conserved, suggesting that it has an important role in PLK activity regulation. Together, these data demonstrate that the PLK1 inhibitory effect on the intra-S-checkpoint response is determined by its degradation via SCF^FBXW7α^/proteasome.

During the progression of this work, it has been reported that FBXW7 governs CDH1 activity in a cyclin E-dependent manner, indicating that loss of FBXW7 increases expression of APC/C^CDH1^ substrates, PLK1 among them [47]. These results raise the possibility that the degradation of PLK1 via FBXW7α that we report may be indirect and that CDH1 is indeed responsible for this degradation. However, a number of arguments support that FBXW7α directly governs PLK1 levels. First, PLK1 is only an APC/C^CDH1^ substrate between late anaphase and G1, or in response to genotoxic stress in G2, since the APC/C^CDH1^ is only active during this period [26, 27]. Second, PLK1 and FBXW7α are both located in the nucleus (Fig 1C), whereas CDH1 is located in the nucleus during G1 but redistributes to the cytosol between S phase and the end of mitosis [48]. Third, APC/C^CDH1^ mediates proteasomal degradation of the ubiquitin-conjugating enzyme UbcH10, providing a negative feedback mechanism that inactivates APC/C^CDH1^ during G1/S transition [49]. Fourth, when the replication fork is stalled, APC^CDH1^ activation is prevented by ATR/Chk1 activity-promoted degradation of CDH1 (supplementary Fig S5 and [50]). Therefore, at least during the APC/C^CDH1^ inactivation period, FBXW7 cannot modulate CDH1 activity. Finally, our *in vitro* ubiquitination assays clearly demonstrate a direct ubiquitination of PLK1 by SCF^FBXW7α^. In fact, the PLK1 mutant in the FBXW7 phosphodegron was unable to be degraded by FBXW7 signaling pathway. Hence, we can conclude that PLK1 is a direct target of SCF^FBXW7α^ for degradation via the proteasome.

Prior to our work, multiple SCF^FBXW7α^ substrates had been identified. However, it remained largely unknown how these substrates contribute to the tumor suppression function of FBXW7α. We speculate that PLK1 may contribute to this function. For example, it is known that PLK1 is involved in checkpoint adaptation, a process originally described in *Saccharomyces cerevisiae*, whereby cells can override the checkpoint and resume cycling with damaged DNA if lesions are not repaired or are incompletely repaired. In human U2OS cells, adaptation was promoted by inhibiting Chk1 and delayed by depleting PLK1 [51]. The authors proposed that Chk1 and PLK1 might control the process of adaptation by independent signaling pathways. In human cells, checkpoint adaptation might potentially promote genomic instability and lead to cancer [52]. Based on our proliferation assays using the non-degradable PLK1 mutant, where transfected cells displayed accelerated proliferation following UV irradiation compared with wild-type PLK1, we could predict that human tumors lacking FBXW7α could have enhanced checkpoint adaptation, making this an interesting area for future research. Nevertheless, we cannot forget the essential role of PLK1 in checkpoint recovery by directly targeting multiple DNA damage checkpoint factors and allowing checkpoint-desactivation [53]. Perhaps, tumors lacking FBXW7α, where PLK1 is not degraded, do not block cell cycle reentry after DNA damage, a possibility that warrants further study.

## METHODS

### Plasmids, cloning, point mutation and sequencing

pCDNA3.1-Flag-FBXW7α, p3xFlag-myc-CMV-24-FBXW7αΔF, pCS2HA-βTrCP, pFlag-CMV2-PLK1, pFlag-CMV2-PLK1-KD, and empty vectors have been previously described [36, 54-56]. pINCY-SPK2, pEGFP-N1, and pCW7 and pCW8 were from Thermo Fisher Scientific, BD Biosciences and ATCC, respectively. pCMVHA-FBXW7α, pCMVHA-FBXW7αΔF and pCMVHA-PLK1 were obtained by cloning the corresponding PCR fragments in pCMVHA. HA-PLK1-T214G was constructed using the “QuickChange II XL Site-Directed Mutagenesis Kit” from Stratagene. Sequence of constructs and point mutation was verified on both strands with an automatic sequencer.

### Cell culture, cell synchronization, transient transfections, drugs and UV irradiation

Routinely, HeLa, HCT116, Cos-7, U2OS and HEK293 cells (from ATCC) were grown in Dulbecco's modified Eagle's medium (Lonza) as described [57]. Cells enriched in the different phases of the cell cycle were also obtained as previously described [58] and confirmed by flow cytometry. DNA constructs were transiently transfected by electroporation or using lipid transfection reagents (Lipofectamine (Invitrogen) or Xfect (Clontech)), and 18h or 48h post-transfection, respectively, cells were harvested and lysed. For some experiments, cells were pretreated with the proteasome and calpain inhibitor Ac-LLnL-CHO (LLnL 100μM, Sigma), cycloheximide (CHX 50μg/ml, Sigma), BI2536 (100nM, Selleck Chemicals), caffeine (10mM, Sigma) or UCN-01 (1μM, supplied by the Division of Cancer Treatment and Diagnosis, National Cancer Institute) and harvested at various times. Where indicated, cells were UV irradiated with 30J/m^2^ and harvested 4h later [57].

### Subcellular fractionation and lysis

Subcellular fractionation was carried out as described [59]. Whole cell extracts were prepared at 4°C in 420mM NaCl, 10mM Tris-HCl (pH 7.5), 1% Nonidet P-40 (NP40), 10% glycerol, 1mM PMSF (phenylmethylsulfonyl fluoride), 1μg/ml aprotinin, 1μg/ml pepstatin, 1μg/ml leupeptin and 10μg/ml chymostatin for 20min. Extracts were centrifuged at 20,000 g for 20min and supernatants frozen in liquid nitrogen and stored at -80°C. Protein concentration was determined using the Bradford assay (Bio-Rad). When necessary, extracts were treated with lambda protein phosphatase (λ-PP) [60].

### Small interfering RNA (siRNA) and half-life assays

Cells were interfered with FBXW7α-, CDH1-, ATM- or ATR-siRNA [37, 61, 62] using the Oligofectamine method (Invitrogen) to suppress the expression of endogenous genes. EGFP-siRNA [61] was used as a non-specific control. Cells were harvested 48h post-transfection and reduction of protein levels confirmed by Western blotting.

Half-life experiments were performed by interfering cells with FBXW7α-siRNA or EGFP-siRNA and, 48h later, adding cycloheximide to the medium. Cells were harvested at the indicated times.

### Electrophoresis, Western blot analysis and antibodies

Proteins were separated by SDS-polyacrylamide gel electrophoresis (SDS-PAGE) and gels were electroblotted onto nitrocellulose membranes and probed with the following antibodies: anti-HA-peroxidase monoclonal antibody (Roche); anti-Cdc25A, anti-cyclin E and anti-p53 monoclonal antibodies, and anti-MCM7 and anti-Grb2 polyclonal antibodies from Santa Cruz; anti-α-Tubulin, anti-Flag and anti-β-actin monoclonal antibodies from Sigma; anti-GFP polyclonal antibody (Immune Systems Ltd.); anti-MCM2 polyclonal antibody (Cell Signaling); anti-PLK1 and anti-CDH1 monoclonal antibodies from Millipore; anti-Nbs1 polyclonal antibody (Novus); anti-Myc monoclonal antibody (Clontech); and anti-Mre11 monoclonal antibody (Gene Tex). Peroxidase-coupled donkey anti-rabbit IgG and sheep anti-mouse IgG were obtained from GE Healthcare. Immunoreactive bands were visualized using the Enhanced Chemiluminescence Western blotting system (ECL, GE Healthcare).

### *In vitro* kinase assays

Anti-HA immunoprecipitates from HCT116 cells transfected with pCMVHA-PLK1, pCMVHA-PLK1-T214G or pCMVHA were incubated with α-casein dephosphorylated (Sigma), (γ-^32^P) ATP and a kinase buffer (50mM Tris-HCl (pH 7.5), 10mM MgCl_2_, 0.1mM Na_3_VO_4_, 2mM DTT and 100μM unlabeled ATP) for 15 min at 30°C. Reactions were terminated by adding 4x SDS-sample buffer and proteins analyzed by SDS-PAGE and autoradiography.

### Co-immunoprecipitation experiments

Nuclear extracts or whole cell extracts diluted to 150mM NaCl (1-2 mg) were incubated with normal mouse serum (Santa Cruz) for 30 minutes and subsequently with protein G-sepharose beads (GE Healthcare) for 1 hour at 4°C. After centrifugation, beads were discarded and supernatants incubated for 2 hours with anti-PLK1 (Millipore) or anti-Flag (Sigma) monoclonal antibodies or normal mouse serum, followed by protein G-sepharose beads for 1 hour. Beads were washed and bound proteins were solubilized by the addition of SDS-sample buffer heated at 95°C for 5 minutes.

For immunoprecipitation to *in vitro* kinase assays, an anti-HA monoclonal antibody from Covance was used.

### Protein identification by liquid chromatography-tandem mass spectrometry (LC-MS/MS)

Flag-FBXW7α was transfected into Cos-7 cells and, after 18h, cells were treated for 4h with LLnL and FBXW7α immunoprecipitated from nuclear extracts with anti-Flag previously coupled to a resin from Pierce (AminoLink Plus Coupling Resin). Resins were washed three times in NP-40 lysis buffer and six times in ammonium bicarbonate 50mM. Samples were analyzed by LC-MS/MS using a LTQ mass spectrometer (Thermo Electron) as described previously [63].

### Immunofluorescence microscopy

U2OS cells were grown on coverslips, fixed with 4% paraformaldehyde and permeabilized with 0.1% Triton X-100. Immunostaining, using monoclonal anti-PLK1 (Millipore) and polyclonal anti-FBXW7α (Zymed) antibodies, and counterstaining with DAPI to visualize the nuclei, was carried out according to standard protocols. Epifluorescence microscopy was performed using a Leica microscope.

### Chromatin fractionation

The soluble fraction of cells was prepared by lysis in CSK buffer (10mM PIPES pH 6.8, 100mM NaCl, 300mM sucrose, 3mM MgCl_2_, 1mM EGTA, 1mM DTT, 1mM PMSF, 50mM NaF, 0.1mM Na_3_VO_4_, 20mM sodium pyrophosphate, 0.5% Triton X-100, 1μg/ml aprotinin, 1μg/ml pepstatin, 1μg/ml leupeptin and 10μg/ml chymostatin) for 5 min on ice. Cell lysates were centrifuged at 7,500 rpm for 5 min at 4°C, and the supernatants collected and clarified by high-speed centrifugation (10 min at 14,000 rpm, 4°C). The chromatin pellet was washed again with CSK buffer and centrifuged at 7,500 rpm for 5 min at 4°C. For nuclease digestions, chromatin pellets were resuspended in lysis buffer containing 50 units of RNase-free DNase I (Roche) and 1mM CaCl_2_ and then incubated at 37°C for 30 min, followed by chilling on ice and centrifugation. The DNase pellet was washed once in 1ml of ice-cold CSK buffer (5 min, 14,000 rpm, 4°C) and incubated in ice-cold CSK buffer containing 500mM NaCl for 10 min at 4°C. This extract was clarified by centrifugation (10 min, 14,000 rpm, 4°C) and pooled with the DNase-treated fraction, constituting the chromatin-associated fraction. After the protein concentration was adjusted, proteins were resolved by SDS-PAGE and analyzed by Western blotting.

### *In vitro* and *in vivo* ubiquitination assays

The *in vitro* ubiquitination of PLK1 was performed in a volume of 10μl containing 50mM Tris-HCl (pH 7.6), 5mM MgCl_2_, 0.6mM DTT, 2mM ATP, 2μl *in vitro* transcribed/translated unlabeled F-box protein, 1.5ng/μl E1 (His_6_-ubiquitin activating enzyme, Boston Biochem), 10ng/μl His_6_-UbcH3 (E2, Boston Biochem), 10ng/μl UbcH5a (E2, Boston Biochem), 2.5μg/μl ubiquitin (Sigma), 1μM ubiquitin aldehyde (Boston Biochem), and 1μl ^35^S-methionine-labelled *in vitro* transcribed/translated PLK1 as substrate. The reactions were incubated at 30ºC for 1h and analyzed by SDS-PAGE and autoradiography. For some experiments the unlabeled F-box protein was substituted by a recombinant SCF^FBXW7α^ complex expressed in Sf21 insect cells.

The *in vivo* ubiquitination experiments were performed in Cos-7 cells transfected and treated with LLnL 4 hours before harvesting. Cells were washed in PBS, lysed at 95ºC for 15 minutes in NP40 buffer supplemented with 5% SDS and 10mM iodoacetamide, and diluted in NP40 buffer supplemented with 10mM iodoacetamide. PLK1 was immunoprecipitated and proteins separated by SDS-PAGE, electroblotted and probed with different antibodies.

### Proliferation assays

Transfected HeLa cells were arrested in S phase with hydroxyurea (0.5mM) for 24h and, after releasing, cells were untreated or irradiated (30J/m^2^) and counted at different time points. Adherent cells were trypsinized, collected and viable cells (trypan blue-excluding) were counted using a hemocytometer. In parallel, as another method for measuring cell proliferation, the protein amount was quantified using the Bradford assay (Bio-Rad).

## SUPPLEMENTARY FIGURES


