# Supplementary material for: SCFFBXW7α modulates the intra-S-phase DNA-damage checkpoint by regulating Polo like kinase-1 stability
Source: Oncotarget. 2014 May 27;5(12):4370–83. doi: 10.18632/oncotarget.2021 (PMC4147330; doi:10.18632/oncotarget.2021)
Supplement: Supplementary file 1 [file oncotarget-05-4370-s001.pdf]

SCF$^{FBXW7\alpha}$ modulates the intra-S-phase DNA-damage checkpoint by regulating Polo like kinase-1 stability

Supplementary Material

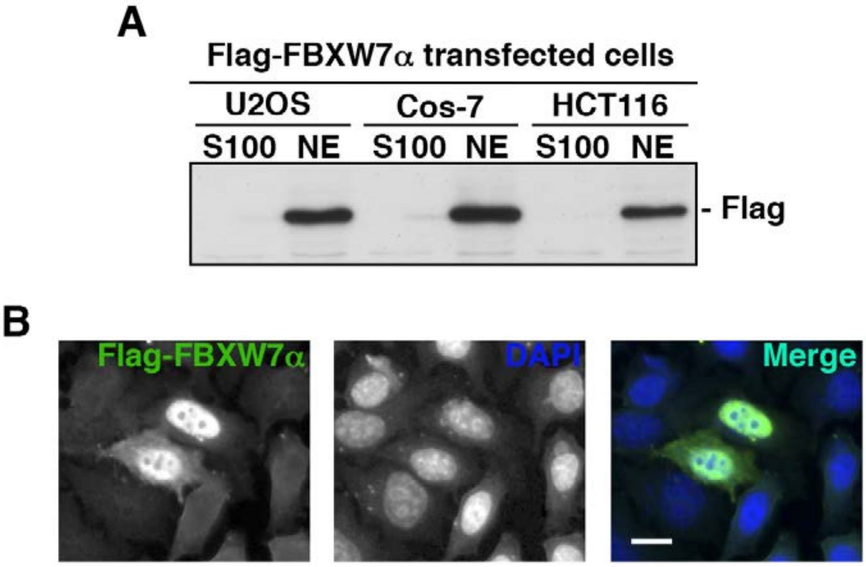

Figure S1: Flag-FBXW7α is located in the cell nuclei. (A) Cytosolic (S100) and nuclear extracts (NE) from different cells transfected with pCDNA3.1-Flag-FBXW7α were analyzed by Western blot. (B) HeLa cells were transfected with pCDNA3.1-Flag-FBXW7α and plated on glass coverslips for immunofluorescence microscopic analysis. Mouse anti-Flag antibody was used to detect transfected FBXW7α and DAPI to stain the nuclei. In the merge, Flag-FBXW7α staining is shown in green and DAPI in blue. Bar, 10μm.

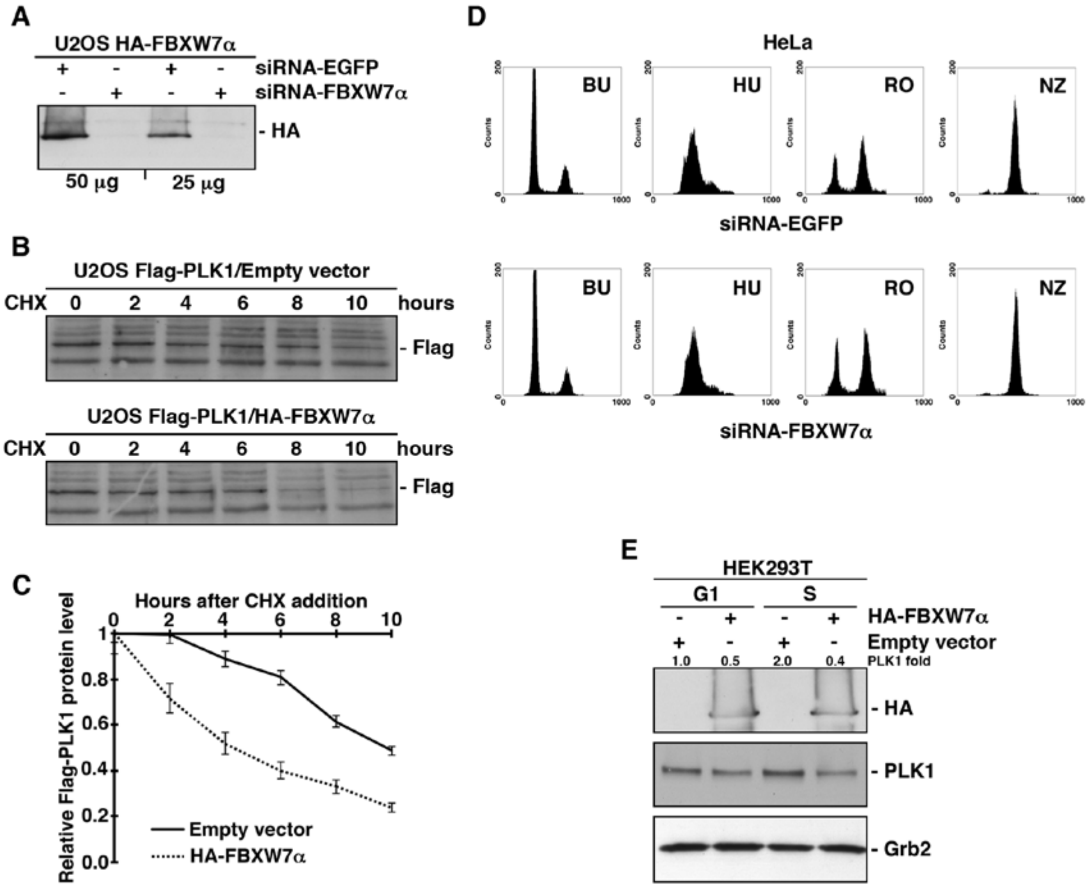

Figure S2: (A) U2OS cells were transfected with pCMVHA-FBXW7α and interfered with the indicated siRNA. After 48h, 25 and 50µg of proteins from lysates were analyzed by Western blotting. (B) Half-life of Flag-PLK1 in U2OS cells cotransfected with pCMVHA-FBXW7α or pCMVHA (empty vector). After 18h of expression, cycloheximide (CHX) was added to the medium and cells were collected at the indicated times. Flag-PLK1 was detected by immunoblotting with anti-Flag monoclonal antibody. (C) Quantification of Flag-PLK1 protein levels presented in (B) using the ImageJ software. Error bars represent the S.D. (n=3). (D) HeLa cells were interfered with the indicated siRNA, arrested in the different phases of the cell cycle, and analyzed by flow cytometry. BU, butyrate; HU, hydroxyurea; RO, RO-3306; NZ, nocodazole. (E) HEK293 cells were transfected with pCMVHA-FBXW7α or pCMVHA (empty vector) and cell synchronization carried out as described. Extracts were analyzed by Western blot.

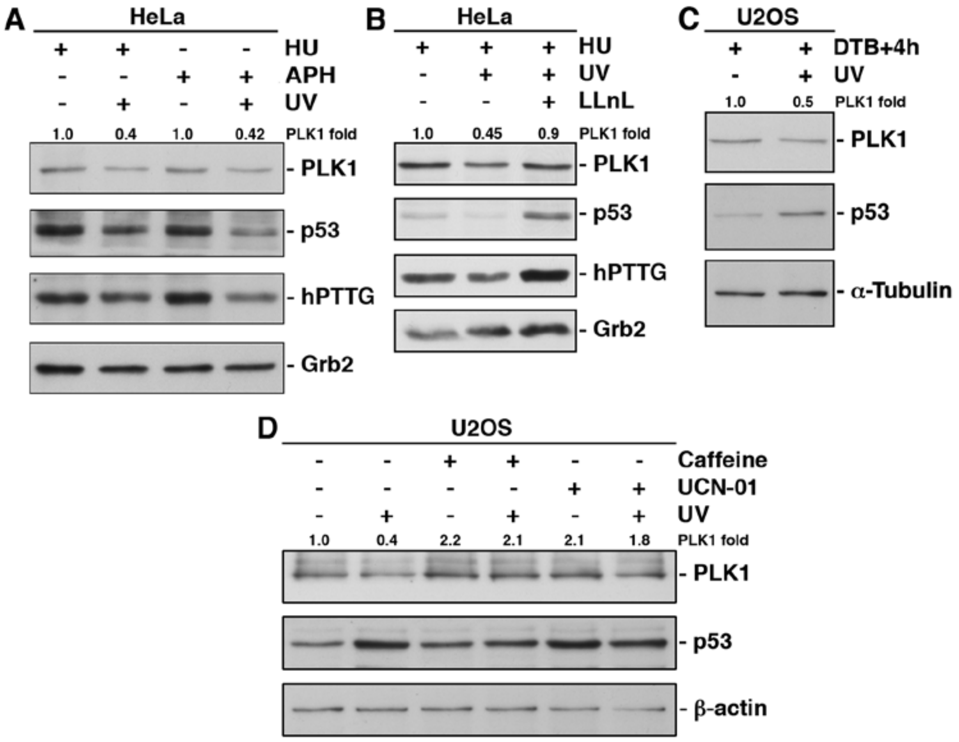

Figure S3: UV irradiation induces PLK1 degradation in S phase. (A) HeLa cells were arrested in S phase with hydroxyurea (HU) or aphidicolin (APH), irradiated (30J/m^2) or not, and harvested 4h later. Extracts were analyzed by immunoblotting. (B) HeLa cells were arrested as in (A) and, where indicated, treated with LLnL 30min before irradiation. Extracts were also analyzed by Western blot. (C) U2OS cells were arrested at the G1/S boundary by double thymidine block (DTB), released into fresh medium, and harvested 4h later. Irradiated cells were subjected to 30J/m^2 in S phase and collected 4h later. Extracts were analyzed by immunoblotting. (D) U2OS cells were arrested in S phase with hydroxyurea and treated with caffeine or UCN-01 30min before irradiation (30J/m^2). Cells were harvested 4h later and extracts analyzed by Western blot. The quantitative fold change in PLK1 was determined relative to the loading control.

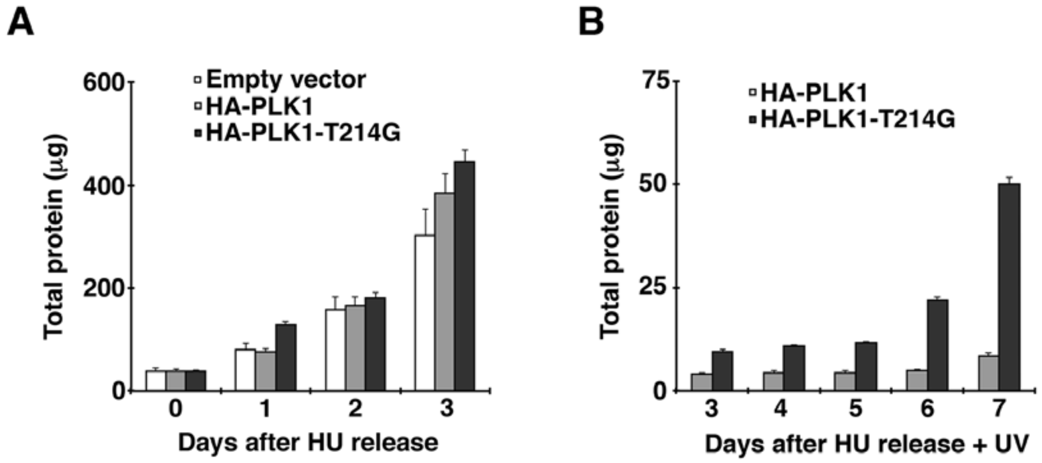

Figure S4: (A) HeLa cells were transfected with pCMVHA (empty vector), pCMVHA-PLK1 and pCMVHA-PLK1-T214G for 18h, arrested in S phase with HU for 24h and, after releasing, harvested at the indicated times. Whole cell extracts were prepared and proteins quantified by the Bradford assay. The analysis began with equal protein amount of the differently transfected cells. Error bars represent the S.D. (n=3). (B) Transfected cells were treated as in (A) but just after HU release, cells were UV irradiated. The protein amount was quantified, starting three days after release, for 5 days.

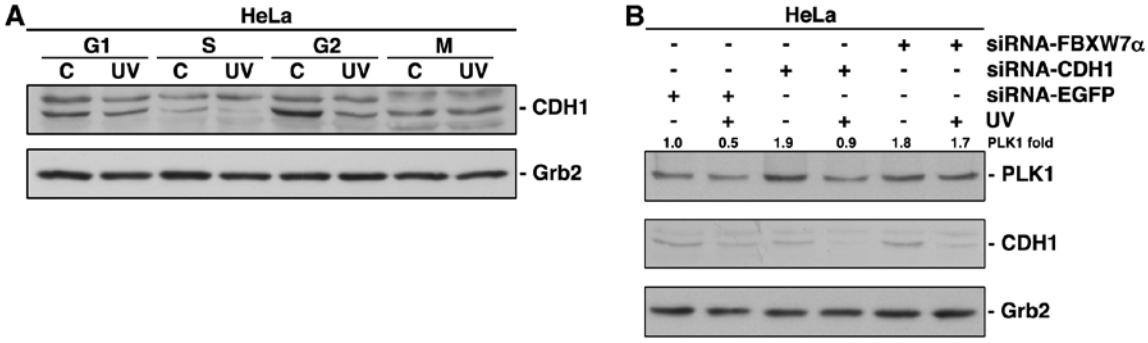

Figure S5: CDH1 is degraded after UV irradiation in S phase and is not involved in PLK1 degradation in these conditions. (A) HeLa cells were arrested in the different phases of the cell cycle and irradiated (30J/m^2) 4h before harvested. Whole cell extracts were blotted with anti-CDH1. Grb2 expression was used as a loading control. (B) HeLa cells were interfered with the indicated siRNA, arrested in S phase with hydroxyurea and irradiated (30J/m^2) where indicated. Extracts were analyzed by Western blot. The quantitative fold change in PLK1 was determined relative to the loading control.
